# Investigating how Microglia Fuel their Immune Responses in Alzheimer's Disease

**DOI:** 10.1002/alz70855_097498

**Published:** 2025-12-23

**Authors:** Amy F Lloyd

**Affiliations:** ^1^ University of Dundee, Dundee, Angus, United Kingdom

## Abstract

**Background:**

Chronic inflammatory microglia is a key pathological hallmark of Alzheimer's Disease (AD) requiring high metabolic demand. Glucose is a key metabolite vital for microglia responses, and sustained hyperglycaemia is a significant risk for AD development. Understanding how microglia fuel their inflammatory responses is key to understanding how to modulate their behaviour in neurodegenerative diseases.

**Method:**

Microglia were isolated from APP‐NL‐G‐F mice at different key time points in disease progression (1, 3 and 6 months, *n* = 4‐6 per time point) and interrogated using mass spectrometry and proteomic analysis.

**Result:**

Proteomic analysis revealed by microglia massively upregulate their glycolysis machinery at 6 months coinciding with significant increases in inflammatory and disease associated protein expression. Interestingly, expression of glucose receptors were significantly decreased, yet increases in proteins involved in glycogen breakdown were increased. Acute (24hr) depletion of glucose *in vitro* also had no effect on microglia inflammatory responses to lipopolysaccharide (LPS) and glycolysis was maintained, yet longer (48hr) depletion significantly altered the microglia response. This suggests that microglia rely on glycogen reserves for metabolic flexibility and to fuel their inflammatory responses.

**Conclusion:**

Microglia utilise their internal stores of glycogen to fuel their inflammatory responses to amyloid (in vivo) and to LPS (*in vitro*). Glycogen regulated microglia responses may represent a novel therapeutic strategy for targeting microglia inflammation in neurodegenerative diseases.

